# Characteristics of Older Adults Seeking Hearing Aids for the First Time and Initial Fitting Parameters in Mainland China

**DOI:** 10.3390/audiolres16030063

**Published:** 2026-04-23

**Authors:** Lena L. N. Wong, Sin P. Lai, Elaine Ng, Alessandro Pasta, Asterios Nastas

**Affiliations:** 1 Clinical Hearing Sciences (CHearS) Laboratory, Faculty of Education, University of Hong Kong, Hong Kong; 2 Demant A/S, 2765 Smørum, Denmark; ehon@oticon.com (E.N.); asteriosnastas@gmail.com (A.N.)

**Keywords:** culture, healthcare, hearing aid

## Abstract

**Objectives**: This data-driven study aimed to explore the characteristics and initial hearing aid (HA) fitting parameters among older adults in Mainland China. **Methods**: Data were extracted from Oticon’s internal database, focusing on 82,834 older adults aged 55 or above who sought HAs for the first time. **Results:** Demographic details (e.g., age and gender), hearing-related data (e.g., the severity of hearing loss), and HA parameters (i.e., laterality of fitting, HA style, earpieces, gain settings, directionality settings, and noise reduction settings) were analyzed. The mean age was 71. There were more males (54.7%) than females, and the majority (78.1%) had at least moderately severe hearing loss. Bilateral fittings were common (76.6%), with receiver-in-canal (RIC) HAs being the dominant style (80%) and open fittings prevalent (44.1%). HA gain was set to below prescribed targets, along with adaptive directionality (93.4%) and low noise reduction levels (>68%). **Conclusions:** These findings offer insights into the Chinese hearing healthcare market. Future research should incorporate data from follow-up sessions to provide a more comprehensive understanding of the landscape, such as adjustments needed after initial fitting after first-time users have spent some time adapting to the use of HAs.

## 1. Introduction

According to the World Health Organization [1], approximately 1.5 billion people globally experience some form of hearing loss (HL), with about 430 million requiring rehabilitation for diminished hearing capacity. This trend is exacerbated by demographic shifts due to an expanding global aging population and increasing life expectancy, suggesting that the global prevalence of HL could rise to around 2.5 billion, with 700 million individuals requiring hearing rehabilitation by 2050. Notably, around 80% of those affected by moderate-to-profound HL reside in low- and middle-income countries, such as Mainland China.

Mainland China faces a pronounced HL burden, evident from a significant surge in the older population over the past two decades. According to the Seventh National Population Census [2], individuals aged 60 and above constitute 18.7% of the total population. With China undergoing one of the fastest rates of population aging globally, the number of individuals affected by HL skyrocketed from 109 million to 407 million from 1999 to 2019 [3]. The Report on Hearing Health in China (2025) revealed that 11.04% of adults aged 60 and above exhibit hearing loss, and this affects over 20 million people [4]. By 2034, the older adult population is projected to reach a staggering 561 million, making up approximately 40% of the total population. Chinese older adults typically acquire HAs when their hearing loss progresses to at least a moderate to severe degree [5,6,7,8], but despite the growing needs, the HA uptake rate is low, estimated at less than 10% in Mainland China [6,9,10]. There is, thus, an urgent need for comprehensive strategies to address the growing burden of HL in China.

### 1.1. Cultural Barriers to HA Uptake That Differentiate Chinese Older Adults

Like their counterparts in Western societies, Chinese older adults regard HAs an inevitable and normal process of aging [11,12,13] but have concerns over the stigma that HAs are a visible sign of disability, as well as self-image and negative judgments about their competence [14,15,16], which may discourage HA uptake. At the same time, older adults may avoid HAs as they may not recognize the need for intervention, opting instead for alternative coping strategies such as increasing the volume on televisions and relying on others to accommodate their hearing difficulties by speaking louder or repeating themselves.

### 1.2. Effects of Healthcare Infrastructure on HA Fitting

The lack of public funding and trained personnel may cause inconsistencies in the quality of healthcare. The cost of HAs is borne by the users or family [17]. However, according to the Fourth National Survey on Elderly People in Urban and Rural China, the average per capita income of urban and rural older adults in 2014 was about CNY 23,900 per year and CNY 7620 (about USD 3900 and 1200) per year [18], respectively, not only limiting the technologies they can afford but also delaying uptake to when they are absolutely needed.

There are few hearing care professionals in Mainland China, exacerbating the accessibility gap in hearing care and reducing the quality of hearing aid fittings. As there are about 20 institutions offering audiology training, hearing aid fittings are mainly carried out by dispensers [17,19], with Wong et al. reporting that 58.4% are not certified, while 18.6% have less than one year of experience, and 45.7% have 1 to 5 years of experience [20].

### 1.3. The Need to Examine First-Time Hearing Aid Fitting Parameters

In order to understand the current hearing aid adoption situation in Mainland China, we examined hearing aid fitting parameters and patient demographics in the database of a hearing aid manufacturer. The data were reported by seniors who reported hearing aid use for the first time or by their families on their behalf. The aims of this study were as follows:

1. To examine the demographics of older adults considering hearing aids for the first time. Knowing this will help us target older adults and reach out to those who have not sought help.
2. To analyze the hearing aid fitting parameters used in initial trial sessions.

Based on previous findings [16,21,22,23,24], we expected these older adults to have more than moderate bilateral hearing loss and thus require hearing aid fitting parameters that would be consistent with this degree of hearing loss.

## 2. Methods

### 2.1. Study Design

For the current study, data were extracted from the internal database of Oticon A/S (Smørum, Denmark), which contains anonymized data without personal identifiers, logged demographic characteristics, and HA fitting parameters from hearing care practitioners worldwide, who utilize Oticon’s fitting software (Genie 2, Oticon A/S, Smørum, Denmark) and maintain an active Internet connection. No participant recruitment was conducted as all data were extracted from existing records. This study was reviewed and approved by the Human Research Ethics Committee (HREC) of the University of Hong Kong. A consent waiver was granted as the data was anonymized.

### 2.2. Study Sample and Variables

Data from initial HA fitting sessions in Mainland China from 2023 were extracted from the database. The inclusion criteria were as follows: individuals (1) aged between 55 and 100 years old; (2) who had no previous experience with HAs (experience level recorded as “none” in the fitting software); and (3) whose pure-tone average air-conduction thresholds at 0.5, 1, 2, and 4 kHz in the aided ear(s) exceeded 25 dB HL but were no more than severe HL. Degrees of HL were categorized as mild (26 to 40 dB HL), moderate (41 to 55 dB HL), moderately severe (56 to 70 dB HL), and severe (71 to 90 dB HL), according to Clark [25].

The variables extracted from the database included demographic factors, audiometric information, fitting modalities, and HA settings. Demographic variables consisted of age and gender, while audiometric data included the degree of HL and hearing thresholds from 250 to 8k Hz. Fitting modalities covered the laterality of HAs (unilateral or bilateral fitting); HA styles classified as behind-the-ear (BTE), receiver-in-canal (RIC), and custom (ITE, ITC, CIC, IIC); earpieces such as instant-fit domes and custom earmolds; and vent sizes of the earpieces. Lastly, HA settings included basic frequency-gain prescription, noise reduction (NR) levels in simple and complex listening environments, and directionality.

The adaptation manager was designed to gradually increase gain and adjust the frequency response from an initial comfortable setting to the prescribed target, ensuring smooth acclimatization for the user. It comprises three adaptation levels: Level 1 provides less overall gain and a flatter frequency response to promote high first-fit acceptance; Level 2 serves as an intermediate step; and Level 3 applies the full prescribed fitting targets. Directionality settings included an adaptive microphone, omni-directionality, and full directionality. The NR function reportedly scans the input acoustic signal 500 times per second, mapping different sound sources and estimating the signal-to-noise ratio (SNR) and noise level in each channel [26,27]. These parameters indicate the adversity of the listening environment. Specifically, the SNR, which ranges from −10 to +15 dB, serves as the primary determinant in classifying the environment as simple or complex and in predicting noise attenuation levels [27]. Using default settings and the HAs in a typical sound environment, an SNR of about –5 dB is typically applied to differentiate between simple and complex listening environments. We adopted this SNR value in our analysis and interpretations. The fitting software had six default NR attenuation settings for personalization, three for each listening environment. In simple listening environments, such as a quiet living room and a family dinner setting with an SNR higher than −5 dB, NR strengths were classified as low level (−0 dB), medium (−2 dB), or high (−4 dB). In complex listening environments such as restaurants and busy streets with an SNR lower than −5 dB, NR strengths were classified as low (−6 dB), medium (−8 dB), or high (−10 dB).

## 3. Results

Data manipulation and analysis were performed in Python 3.10 (in the Databricks environment). Records from a total of 82,834 individuals were identified from the database, encompassing 146,304 ears fitted with HAs. Patients were selected only if they met all the criteria stated above.

**Figure 1:**
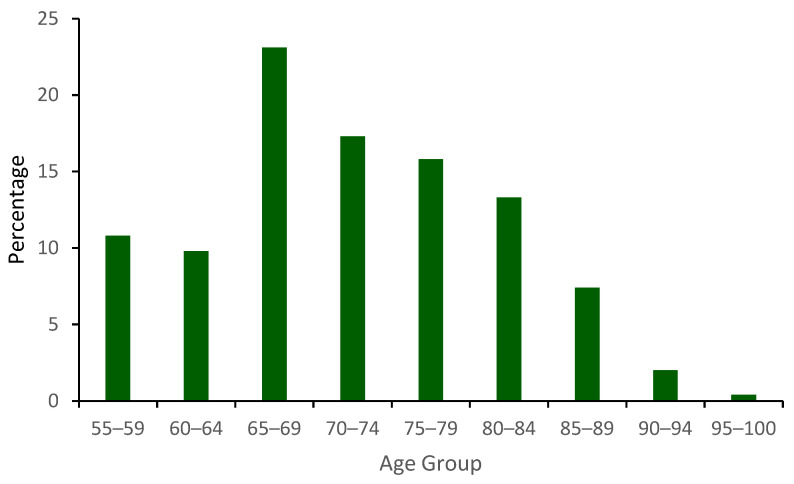
Age distribution.

Demographic variables were analyzed based on individual data. The mean age was 71.9 years (SD = 9.18), with the highest proportion falling within the 65–69 age group (23.1%), followed by the 70–74 age group (17.3%) and the 75–79 age group (15.8%). There was a gradual decline in the number as age increased (see Figure 1). In terms of gender distribution, there was a higher proportion of males (54.7%) compared to females (40.1%); gender information was not available for others. Bilateral fittings accounted for 76.6% of the individuals.

**Figure 2:**
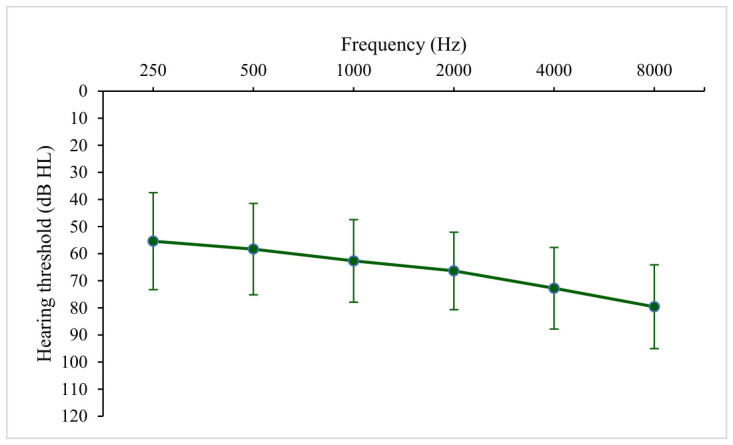
Means and standard deviations of hearing thresholds of aided ears (*N* = 146,304).

Table 1 presents the hearing characteristics and initial fitting parameters of the fitted ears. Among them, 47.7% exhibited moderately severe loss, followed by severe loss (30.4%). On average, users demonstrated sloping, moderately severe to severe loss across frequencies from 250 to 8k Hz (see Figure 2). Identical audiometric thresholds between ears were found in 70% of individuals.

Hearing aid fitting parameters were analyzed based on ears fitted. Among the hearing aid fitting parameters, there was no missing data concerning the style of the hearing aids or earpieces; there was 0.05% missing data for noise reduction settings, directionality, and adaption level.

Over 80% of the fitted ears received RIC fittings, with 44.1% of them being prescribed with double-vented domes featuring a vent size of 1.4 mm. Most were prescribed with adaptation level 1, where the gain was lower than the prescribed target. Nearly all (93.4%) were fitted with adaptive microphones. In simple listening environments with an SNR greater than −5 dB, a low NR strength (−0 dB) was the most commonly prescribed (68.2%). In complex listening environments with an SNR lower than −5 dB, a low NR strength (−6 dB) was again the most frequently prescribed (68.3%).

## 4. Discussion

The current study is the first to examine hearing aid fitting practices for first-time hearing aid seekers in Mainland China. We discuss the findings considering influences from culture and hearing care infrastructure, although the information often was not derived directly from research on audiology or hearing aid fitting.

### 4.1. Age

The average age was approximately 71 years, and there were more individuals between 65 and 74 compared to those aged 75 or above. This trend is consistent with research from Taiwan, which found a negative correlation between HA adoption and age among Chinese older adults aged 65 to 90 [28]. Although studies from Western societies have shown that older individuals were more inclined to adopt HAs with an increased HL severity and that there is reduced stigma with increasing age [29,30,31,32], this pattern was not observed in this study. These results should be interpreted in light of the age distribution of the general population in Mainland China. Specifically, the younger age group (65 to 74 years old) represents a larger segment of the population (9.61%) compared to the older age group (75 years and above), which accounts for 5.27% [2]. Additionally, the severity of HL combined with the more active social participation of younger seniors could contribute to the higher percentage of this population in the demographics of this study.

#### Effects of Chinese Culture and Lifestyle

Wu and Sheng [33] reported that younger Chinese seniors (aged 60–74) tend to have more active social lives with friends and neighbors after retirement than older seniors, actively engage in caregiving of their grandchildren, and frequently travel [34]. Their larger social networks and higher rates of social interaction might make younger seniors more aware of their hearing loss and thus more willing to use HAs when daily activities and communication are impacted. Moreover, younger seniors generally have higher educational levels, with the majority having at least a primary school education, compared to older seniors, most of whom did not finish primary school education or are illiterate [35]. Individuals with higher education levels typically have a better understanding of health-related information and are more adept at following healthcare practitioners’ recommendations for managing their health. For example, they can accurately comprehend health advice, which increases the effectiveness of medical treatment [36,37]. As a result, they may have greater awareness of the benefits of HAs and a higher likelihood of adopting them.

In contrast, older seniors aged 75 or above are more susceptible to social isolation due to age-related life changes such as the death of a spouse [33,38]. Spouses play a vital role in raising awareness of HL and encourage help-seeking behavior and HA adoption [31,39,40]. They become increasingly crucial in providing support, especially when other support networks are lacking [33,41]. Thus, the loss of a spouse could result in reduced communication and less frequent feedback about hearing difficulties, potentially leading to the normalization or neglect of hearing problems, even in severe HL cases. These older seniors may have diminished support networks and live alone, decreasing the willingness to use HAs [28,42].

Although traditionally, the Confucian value of filial piety, emphasizing adult children as the primary supporters of their parents, has long influenced the beliefs of Chinese older adults, particularly older seniors [33,43], societal changes have weakened family relationships. The one-child policy has reduced family sizes, and urbanization has encouraged adult children to move away for work [43,44]. As a result, the living arrangements of Chinese older adults have shifted from traditional multigenerational households (64.9% in 2000 to 48.75% in 2015) to empty-nesting (the proportion of empty-nesters increased from 35.1% to 56.3%) [35], reducing communication needs.

### 4.2. Gender

In this study, there was a higher proportion of male first-time HA users compared to females, which is consistent with previous research findings [28,40,45,46]. One contributing factor to the higher HA adoption rate among men in China is that males might be more likely to engage in full-time jobs with substantial demands and thus require better hearing sensitivity, prompting them to seek amplification more frequently than females [40].

Traditional Chinese family roles, where men are primarily responsible for financial provision while women focus on caregiving for older family members and children, have long influenced Chinese trends. This cultural expectation has historically constrained the women’s participation in the labor force [47]. According to data from the World Bank, in China in 2024, the labor force participation rate was significantly higher for men, at 71.1%, compared to 59.6% for women [48]. During the working years of the current older adult population, the economy of China was predominantly driven by agricultural and industrial sectors due to rapid industrialization [49]. As indicated by the limited or primary-level education of the majority of Chinese older adults [18], many had worked in these sectors, which often led to them being exposed to hazardous noise levels without adequate occupational health precautions [50]. Over 80 million Chinese workers were estimated to have been exposed to hazardous noise levels [51]. Men, who are more likely to work in noisy environments [46,52], are particularly susceptible to noise-induced HL. Zhou et al. [53] found that over 30% of Chinese male workers suffer from high-frequency HL due to prolonged exposure to hazardous noise levels. The gender disparity observed in this study also aligns with the higher prevalence of HL among males compared to females in China [54].

### 4.3. Typical Hearing Sensitivity

Our study found that most first-time HA users exhibited moderately severe HL, with the average hearing thresholds ranging from moderate (55.35 ± 17.90 dB HL) at 250 Hz to severe (79.56 ± 15.45 dB HL) at 8k Hz. These results are similar to those of previous research suggesting that Chinese older adults often delay getting HAs until their HL reaches a moderate or severe stage [7,55]. Dispensers have also reported the majority of their patients (60%) possess a 61 to 70 dB HL [20]. A few other studies have also reported that they might not perceive significant hearing difficulties until their HL exceeds 45 dB and that HAs are typically adopted when the loss reaches 65 dB [5,28].

In addition to the factors mentioned in the Introduction, this delayed recognition of HL may be attributed to the linguistic characteristics of tonal languages. Chinese is a tonal language, where the pitch or tone of a word plays a crucial role in conveying its meaning. Within this linguistic framework, the same syllable uttered with different tones can yield entirely different word interpretations. For example, “ma” can signify “mother” with tone one, “hemp” with tone two, “horse” with tone three, and “scold” with tone four in Mandarin [56]. This tonal variation, intertwined with stress and rhythm, is typically encoded within fundamental frequencies, predominantly situated below 500 Hz. These lexical tones crucially contribute to speech understanding in noise [57,58] and might have contributed to the delay in hearing aid adoption.

### 4.4. Symmetry in Hearing Sensitivity

The symmetry in audiometric thresholds between ears suggests these are likely “quick fits” to give first-time hearing aid seekers a glimpse of amplification, rather than customized fittings to optimize hearing. That is, dispensers might have copied and pasted audiometric results from one ear to the other; entered results from soundfield testing, without differentiating the hearing thresholds in the two ears; or entered a “commonly seen” audiogram for patients. The audiograms were used to automatically suggest the default settings for each fitting parameter (i.e., type of earmold, gain, and noise reduction settings). They might have influenced the degree of customization of amplification to optimize outcomes and thus might not provide a good preview. Manufacturers should consider educating their dispensers as well as provide a mechanism in the fitting software to monitor or warn dispensers of the consequences of this practice, when dispensers are prompted by the fitting software to enter audiometric data for each ear.

### 4.5. HA Style and Earpieces

The choice of HA style and earpiece is vital for user satisfaction. In contrast to prior findings suggesting a higher prevalence of seniors being fitted with BTE HAs [6,55], our study indicates a notable shift. Over 80% of Chinese older adults in the database were prescribed with RIC-style HAs, which apparently is consistent with the global shift towards an increasing preference for RIC devices. For example, 81.7% of HAs dispensed in the United States in the first half of 2019 were RIC instruments [59,60,61]. RICs offer a wide fitting range and discreet options for open fitting, enhancing comfort and reducing the occlusion effect, which is commonly reported by HA users with normal to mild low-frequency HL [59,62,63].

Given that the majority of older adults in the current study exhibited low-frequency HL of at least a moderate degree, closed dome would have been recommended by the fitting software. Therefore, it is somewhat surprising that many (44.1%) were prescribed double-vented domes, as well as open domes (13.1%). While previous studies have reported that Chinese speakers complain about occlusion effects [9,10,28], the choices of earpiece and vents in the current study may also reflect hearing care practitioners’ priority in ensuring comfort and acceptance of the new HAs due to their discrete ear tips, wear comfort, and improved esthetics. Previous studies also suggest that Chinese users often complain about muffled sounds, their own voice being overly loud, and excessive amplification at high frequencies [10,28]. These initial hearing aid settings (i.e., low gain, open fittings) are aimed at preventing these issues, and are thus not unexpected.

Despite the benefits of open fittings, they may compromise the amount of gain and the effects of noise reduction. This may lead to reduced speech clarity and potential dissatisfaction with the devices’ performance. The degree of impact can only be ascertained by measuring outcomes with these devices.

### 4.6. Gain Settings

Adequate amplification is crucial to improve access to sounds [64,65]. However, a substantial proportion of those trying hearing aids (87.2%) in this study received gains lower than the prescribed targets based on their audiometric thresholds. Previous research showed that new HA users often prefer lower gain settings compared to experienced users, regardless of the fitting formula utilized [66,67,68,69], and need time to acclimatize to amplified sounds [67].

Many individuals with HL, especially those with presbycusis, experience a gradual, bilateral decline in hearing sensitivity over time and often are not immediately aware of it. As Chinese older adults often wait until their HL reaches a severe degree before purchasing an HA [7,70], they probably have become accustomed to a quieter auditory environment over time. The delay in hearing aid adoption could lead to greater adjustment to hearing background noise, and older adults may thus find amplified sounds initially startling or uncomfortably loud [9,10]. Thus, they may opt for lower amplification levels initially. Consequently, dispensers probably have adapted their fitting strategies to promote initial acceptance of amplification.

Although these settings are not optimal for the degree of hearing loss observed, they are consistent with previous findings concerning hearing aid parameters and outcomes in Chinese speakers (e.g., [8,23]).

### 4.7. Noise Reduction Strengths in Simple and Complex Listening Environments

Directional microphones and noise reduction algorithms could enhance overall listening comfort and speech clarity in noisy environments [26,71,72,73]. The benefits of adaptive directionality have been well documented [74,75]. This feature is often set as the default in many manufacturers’ fitting software, and HA practitioners, such as those in the present study, tend to favor this default setting [76,77]. In fact, over 90% of the fittings in the current study adopted adaptive directional microphones.

Previous research suggests that individuals with quieter lifestyles may prefer comfort-focused settings for prolonged wear. Conversely, people with active social lives may benefit from stronger NR to reduce unwanted noise and improve listening comfort, although users may not always prefer high NR settings due to potential signal distortion, which affects speech intelligibility [78]. Consistent with prior findings, most participants (68.2%) in this study were fitted with 0 dB noise attenuation (i.e., no noise reduction) in simple environments, where higher SNRs and fewer distracting noise sources facilitate speech perception. Conversely, in complex environments with SNRs below −5 dB, the majority (68.3%) were fitted with 6 dB noise attenuation. This preference for approximately 6 dB of attenuation in more challenging listening conditions aligns with the findings of Wong et al. [23], which indicated that most Mandarin speakers preferred some degree of NR, typically around 5 to 6 dB attenuation. However, the HA users in the current study might not have tried these settings in real-life listening environments.

### 4.8. Limitations and Future Directions

This study is the first to report on hearing aid fitting practices in Mainland China using an extensive dataset. Although the results offer valuable insights into hearing healthcare services on the Mainland China, several limitations must be acknowledged and warrant careful consideration when interpreting these data.

#### 4.8.1. Uncertain Hearing Loss

The symmetry of HL in the present study could not be determined due to consistently identical audiometric thresholds between ears; as a result, individual ear analyses were not conducted. Even though it is not uncommon to observe symmetry in presbycusis between ears, findings reported here do not accurately reflect optimal fitting and should be interpreted with care. Dispensers should be warned against entering identical thresholds and be advised of the consequences to precision in hearing aid prescription. Manufacturers should highlight this issue in dispenser training.

Additionally, the dataset only included air-conduction thresholds, making it difficult to identify the specific nature of HL among first-time adopters. The absence of bone-conduction thresholds complicates the distinction between purely sensorineural HL and HL with concurrent conductive components, potentially compromising data quality.

#### 4.8.2. Limited Information

The database utilized in this research only contained age and gender data for first-time adopters, and comprehensive demographic and geographic information was lacking. This limitation restricts our understanding of the user population, making it challenging to segment the user base effectively and identify specific patterns or trends related to different population groups (e.g., socioeconomic status, regional differences).

As we limited our inclusion of hearing loss level to no more than a severe degree, the findings do not apply to those who have profound hearing loss. Exclusion of these participants could have skewed the degree of hearing loss of first-time adopters to a lesser degree. However, due to the significant impact on daily function, older adults are not likely to wait until their hearing loss reaches a profound degree before hearing aid adoption. In other words, these participants probably would not have been included in the study in the first place.

The technology level could have affected the availability of advanced hearing aid parameters and the degree to which extent fitting parameters could have been customized to individual preferences. As information on technology level was not available, the impact could not be evaluated. Despite the majority of fitting parameters being similar across first-time adopters, future studies should examine whether technology levels would make a difference in hearing aid parameter choice and the outcomes.

#### 4.8.3. Uncertain if Findings Can Be Generalized

Interactions among hearing aid parameters influence the overall amplification output, which may not directly reflect individual parameter settings. Here, open fittings may interact with low amplification gain settings to reduce the gain further and may make noise reduction less effective. These effects cannot be ascertained without measuring the outcomes. Premium hearing aids may offer greater flexibilities in hearing aid adjustments compared to basic models. However, in the present study, the data were not analyzed according to HA technology level. It also appears that similar HA settings were applied to most users, raising questions about whether they reflected user preferences or standard practices among dispensers.

As data was collected only when the dispensers had an active Internet connection and used the manufacturer’s fitting software, the dispensing practice could have differed in locations where Internet access was limited. It is also unclear whether the substantial proportion of bilateral fittings reflects actual purchases and willingness to use two HAs or if the data primarily represents HA trials without purchases by users. This introduces significant uncertainty regarding the actual preferences of Chinese older adults. Lastly, the dataset was sourced from a single HA manufacturer, which may limit the generalizability of the findings and introduce potential bias. However, as a number of dispensers also sell hearing aids from other manufacturers and as about 41% of dispensers have been nationally certified, based on findings from Wong et al. (2024), the practice reported here may not be limited to fittings associated with a single manufacturer [20].

Overall, there could be bias in result interpretation, limiting the generalizability of the findings and our understanding of the factors that could contribute to variations in dispensing practice (e.g., the education background or experience of dispensers, accessibility of hearing health services) and user preferences.

#### 4.8.4. Duplicate Records Not Accounted for

Patients could have visited multiple centers to trial hearing aids, resulting in duplicate records. As they were identified by an anonymous identifier assigned by the fitting software, we could not trace the information back to individual patients to identify or remove duplicate records. However, this factor might not have significantly skewed the findings, given the large sample and consistency in findings.

#### 4.8.5. Future Studies Needed to Examine Outcomes

This is the first study that focused solely on initial HA fitting sessions in order to analyze dispensing practices in Mainland China. Descriptive analyses were employed. Future research can extend these findings by measuring how different fitting parameters, demographic variables, and dispenser education levels affect the outcomes. Longitudinal prospective studies can incorporate data from follow-up sessions to offer insights into how HA parameters, including earpieces and settings, are adjusted over time. We have explained the findings based on our understanding of the Chinese culture and hearing care infrastructure, as well as anecdotal reports from dispensers and audiologists. Validation is, however, needed via future studies that systematically examine these variables, their interactions, and outcomes. These studies would enable a better understanding of the evolving preferences and needs of Chinese older adults new to HAs, balancing amplification with comfort and individual preferences.

## 5. Conclusions

This study utilized big data to provide an overview of the characteristics and initial parameters of first-time HA users among Chinese older adults. Our study revealed that Chinese older adults typically acquire their first HAs around the age of 71, when HL reaches a moderately severe degree. Knowing the demographics of older adults who are having hearing aids fitted for the first time could help us cater marketing strategies, patient education, and family outreach. For example, we may want to promote hearing care at venues frequented by the target age groups and design marketing materials to suit their literacy level and activities. With reduced multigenerational living arrangements, independent living may be a theme we can promote.

Bilateral RIC and open fittings were prevalent, with initial gain lower than the prescribed targets, adaptive directionality, and low NR levels for both simple and complex listening environments. It was surprising that, despite the diversity in HL configurations, lifestyles, and preferences, initial fittings for first-time HA users did not vary greatly in the current study. While it is important to examine the outcomes associated with these settings, manufacturers should also consult with their dispensers regarding how they adjust fitting parameters to target future dispenser training towards critical knowledge and skills.

Due to the scarcity of studies that examine first-time hearing aid uptake and factors influencing fitting parameters, we were not able to support our discussion against the literature. Instead, we interpreted the findings against relevant findings on culture and lifestyle in Mainland China. These findings are somewhat speculative and should therefore be validated in future studies that examine their relationships.

Although we did not examine hearing care practices in other countries, we reiterate the need to provide adequate dispenser training in many parts of the world where hearing care is growing. Manufacturers could learn from the problem areas identified in the current study and highlight them in dispenser training. Artificial intelligence could be used to make educational resources available across languages, while government policies regarding dispenser training and certification should be in place to ensure proper practice.

Overall, the current study analyzed current hearing aid fitting practices in Mainland China, finding that dispenser training should be enhanced and personalized fitting should be emphasized.

## Figures and Tables

**Table 1:** Distribution of variables by fitted ears (%) (*N* = 146,304).

Variables	Percentage
Degree of hearing loss	
Mild (26–40 dB HL)	2.1%
Moderate (41–55 dB HL)	19.8%
Moderately severe (56–70 dB HL)	47.7%
Severe (71–90 dB HL)	30.4%
Hearing aid style	
RIC	83.3%
BTE	12.7%
Custom (ITE/ITC/CIC)	4.0%
Earpiece (vent diameter)	
Double-vented dome (1.4 mm)	44.1%
Single-vented dome (0.8 mm)	11.1%
Open dome (2.4 mm)	13.1%
Closed dome (0 mm)	7.7%
Custom mold	24.2%
Gain setting	
Adaptation level 1	87.2%
Adaptation level 2	7.1%
Adaptation level 3	5.7%
Noise reduction strength—simple listening environment	
Low (−0 dB)	68.2%
Medium (−2 dB)	28.6%
High (−4 dB)	3.3%
Noise reduction strength—complex listening environment	
Low (−6 dB)	68.3%
Medium (−8 dB)	28.8%
High (−10 dB)	2.9%
Directionality setting	
Adaptive microphone	93.4%
Full directionality	2.6%
Omni-directional	3.9%

Note. Styles: RIC = receive-in-canal, BTE = behind-the-ear, ITE = in-the-ear, ITC = in-the-canal, and CIC = completely-in-canal. Gain settings: Adaptation levels 1 and 2 = below prescribed target and Adaptation level 3 = at prescribed target.

## Data Availability

Restrictions apply to the availability of these data. Data were obtained from Oticon A/S (Smørum, Denmark) and are available from the authors with the permission of Oticon A/S (Smørum, Denmark).
